# A Spiking Neural Network in sEMG Feature Extraction

**DOI:** 10.3390/s151127894

**Published:** 2015-11-03

**Authors:** Sergey Lobov, Vasiliy Mironov, Innokentiy Kastalskiy, Victor Kazantsev

**Affiliations:** Department of Neurotechnology, Lobachevsky State University of Nizhni Novgorod, 23 Gagarin Ave., Nizhny Novgorod 603950, Russia; E-Mails: mironov@neuro.nnov.ru (V.M.); kastalskiy@neuro.nnov.ru (I.K.); kazantsev@neuro.nnov.ru (V.K.)

**Keywords:** sEMG, feature extraction, pattern classification, artificial neural network, neurointerface, exoskeleton

## Abstract

We have developed a novel algorithm for sEMG feature extraction and classification. It is based on a hybrid network composed of spiking and artificial neurons. The spiking neuron layer with mutual inhibition was assigned as feature extractor. We demonstrate that the classification accuracy of the proposed model could reach high values comparable with existing sEMG interface systems. Moreover, the algorithm sensibility for different sEMG collecting systems characteristics was estimated. Results showed rather equal accuracy, despite a significant sampling rate difference. The proposed algorithm was successfully tested for mobile robot control.

## 1. Introduction

Human machine interface (HMI) development is one of the modern trends in global interdisciplinary sciences and technologies. Generally the HMI is built using biomimetic signals including electroencephalography (EEG) and electromyography (EMG). The HMI is extremely important in the development of control systems for medical rehabilitation devices such as limb prostheses, exoskeletons, training setups, as well as for remote control of autonomous robots and avatars. At present the HMI based only on EEG has a number of limitations related primarily to the need of a large number of recording channels at the input and poor ability to recognize commands on the output. Surface electromyography (sEMG) has advantages in this context.

In the problem of external device control by sEMG one can use several strategies. Simple methods based on single-channel recording permit one to detect thresholds or to implement proportional control in case of continuous monitoring for certain characteristics of the sEMG. Multichannel recording considerably expands the control capabilities based on the sEMG. At the same time, it requires additional signal processing features such as classification (e.g., recognition) of sEMG patterns and multichannel regression proposed in recent studies [1,2]. Pattern classification was combined with command control and, hence, can be used in the case when the control device was equipped with an autonomous, local low-level control system capable of implementing some macro commands.

In streaming classification, one usually does not use row data representing a sensor signal digitized at high sampling frequency. For classification, the sampling rate can be significantly lower than the data acquisition rate. The data stream is segmented into certain time windows, analyzed for each window, and then fed to the classifier. When a large number of features must be detected, different dimension reduction algorithms were applied.

Artificial neural networks (ANN) represent one of the effective tools currently used for pattern recognition in many applications, both as classifiers and for dimension reduction. Moreover, neuron networks can be further implemented in neuro-interfaces providing along with signal processing conditional feedback to the nervous system. In this sense they can be regarded as an artificial continuation or expansion of the nervous system. Note, however, that a natural interface (in terms of analog signal characteristics) between living neurons (projected outside by muscle systems or the peripheral nervous system) requires quite detailed biological models of the artificial neurons used in the ANNs. One of the possible candidates for such models is a spiking neuron providing pulse excitation responding to an incoming stimulus and possessing many important features of living neurons [3]. In recent years researchers have noted the great potential of spiking neurons in many applications [4]. The use of spiking neurons as classifiers in pattern recognition problems of visual information recorded from a silicon retina [5], and in simulation of sound information processing in the auditory cortex [6] has been reported. Evolving spiking networks were also applied for recognition of spatio-temporal patterns [7]. Regarding streaming signal processing, the successful use of spiking neurons was reported in the task of speech recognition [8], in EEG studies with recognition of positive and negative peaks caused by potential P300 [9], as well as in detection of epileptiform conformal activity [10]. 

In this paper we propose an ANN composed of spiking neurons capable of processing sEMG signals with high degree of fidelity compared with existing algorithmic classifiers. Communicating with spikes the system can be integrated with HMI based on the sEMG. The spiking neurons inter-connected with some perceptron-like architecture provide effective feature extraction with classification based on a back propagation error algorithm.

## 2. Models and Methods

The experiments involved 17 healthy research subjects (male and female) aged from 20 to 56 years. The study complied with the Declaration of Helsinki (Finland) adopted in June 1964 and revised in October 2000 (Edinburgh, Scotland) and was approved by the Ethics Committee of Lobachevsky University. All subjects gave their written informed consent.

**Figure 1:**
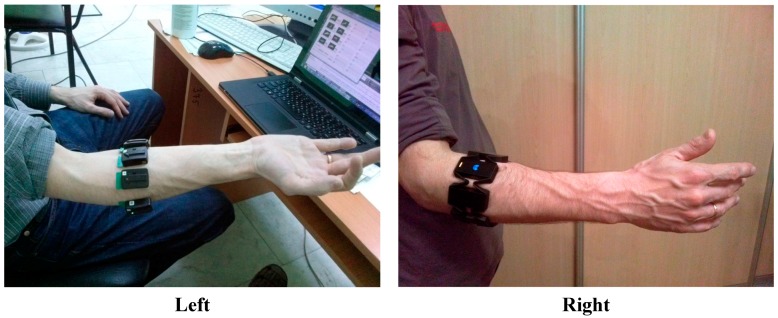
Electrode placement: DELSYS^®^ Trigno™ wireless sensors (**left**) and MYO™ Thalmic bracelet (**right**).

Surface EMG registration was performed using two wireless MYO™ Thalmic and DELSYS^®^ Trigno™ myographic setups. The first device targeted daily use by subjects as a sEMG-based HMI without any special training. To test our algorithms with data collected from a specialized myographic setup we used a DELSYS^®^ Trigno™. The sampling rates in the MYO™ Thalmic and DELSYS^®^ Trigno™ were 400 Hz and 2000 Hz, respectively. We used eight channel mode for signal registration. Location of the electrodes was determined by the form-factor of the MYO bracelet—a ring around the forearm (Figure 1). This placement of the electrodes in some sense complicated the task of the sEMG pattern recognition compared with experiments where the electrode position was guided by localization of motor points of the studied muscles (see, for example, [11]). 

**Figure 2:**
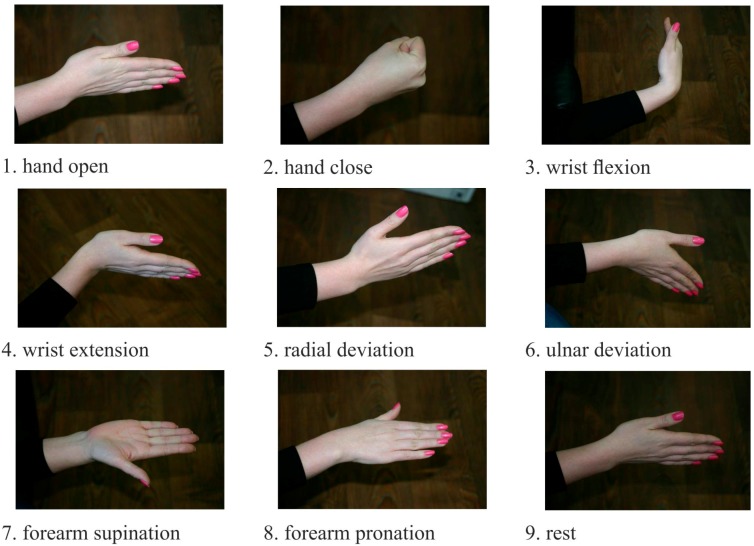
Static gestures, using as motor patterns for EMG pattern recognition.

The classification procedure involves collection of input data in some groups further referred to as classes. At the learning stage the classifier was introduced with a selection of a well-known set of input data and output classes. Then, the recognition process started again with the trained classifier. The goal was to identify certain class among the input data. The sEMG signals were used as the input data, and motor patterns, e.g., static hand gestures, were used as the output (Figure 2). During the experiment, all participants were asked to produce four series of nine gestures lasting 2–3 s in a random order. Between different gestures the wrist relaxed. For training and testing samples recording motor patterns are logged using the graphical user interface of the MyoClass software.

Root Mean Square (*RMS*) of the signals was applied to calculate the muscle contraction amplitude. For each channel, we segmented the data into a window size of 40 samples (100 ms). This cropping was performed with a step size of 20 samples (50 ms). Next, *RMS* for each window was calculated:

(1) $$RMS=\sqrt{\frac{1}{N}\cdot \sum_{n=1}^{N}x_{n}^{2}}$$

where *N* is number of signal values in a time window, *x_n_* is signal value at time *n*.

**Figure 3:**
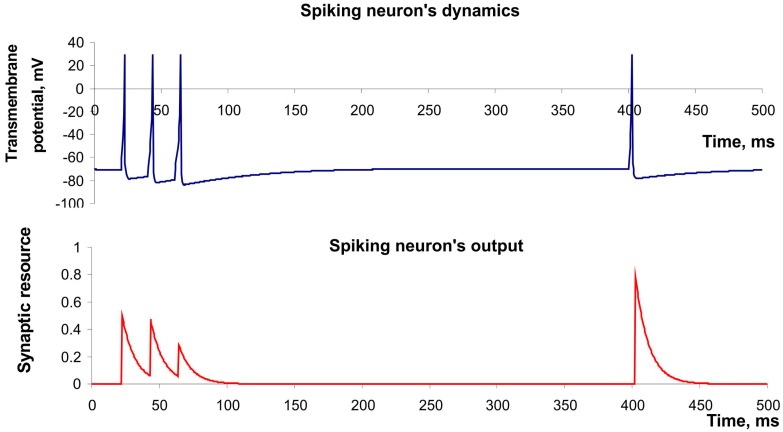
The example of dynamics of spiking neuron described by Izhekevich’s model and its output described by model of Tsodyks and Markram. Parameter values: *a* = 0.02*, b =* 0.2*, c =* −65*, d =* 8, *U* = 0.5, *τ_rec_ =* 100 ms, *τ_I_ =* 10 ms, *τ_facil_* = 1000 ms.

To extract features from the sEMG signal by this approach we apply a pulse neuron model proposed by Izhekevich [12]:

(2) $$\frac{dV}{dt}=0.04V^{2}+5V+140-u+I$$

(3) $$\frac{du}{dt}=a\left(bV-u\right)$$

with the auxiliary after-spike resetting:

(4) if V≥30 mV, then V=c, u=u+d

where *V* accounts for transmembrane potential, *u* is an auxiliary variable, *a*, *b*, *c*, *d* are the model parameters, *I* denotes the current applied to the membrane from the outside. The model can exhibit firing patterns of all known types of neurons with the choice of only four parameters. We used parameters reproducing “regular spiking” neuron dynamics [12]. Figure 3 (upper panel) shows the spike generation of this neuron in response to irregular pulse stimulation. Moreover, Izhikevich’s model has high computational efficiency that is important in design of real-time recognition systems [13].

The synaptic resource (implies a neurotransmitter) was used as the output neuronal signal *y_i_(t)*, while the number of resources released in synapses with each spike was described by the model of short-term frequency-dependent plasticity proposed by Tsodyks and Markram [14]:

(5) $$\frac{dx}{dt}=\frac{z}{\tau_{rec}}-ux\delta \left(t-t_{sp}\right)$$

(6) $$\frac{dy}{dt}=-\frac{y}{\tau_{I}}+ux\delta \left(t-t_{sp}\right)$$

(7) $$\frac{dz}{dt}=\frac{y}{\tau_{I}}-\frac{z}{\tau_{rec}}$$

(8) $$\frac{du}{dt}=\frac{u}{\tau_{facil}}+U\left(\text{1}-u\right)\left(t-t_{sp}\right)$$

where *x*, *y*, *z* denote synaptic resource portion (mediator) located respectively in the reduced, active and inactivated states, *t_sp_* is the time moment of spike appearance, *u* is the variable responsible for the frequency-dependent facilitation/depression, *τ_rec_*, *τ_I_*, *τ_facil_* account for the characteristic times of the synaptic dynamics. The model can describe the decrease of synaptic responses in the case of high frequency spiking (depression) and the increase in the case of low frequency (facilitation). Figure 3 (button panel) illustrates both effects.

Next, we used a multi-layer artificial neural network as the classifier based on the backpropagation learning rule. In a series of preliminary experiments where the *RMS* was used as a feature, parameters of the classifier were chosen to ensure a better recognition accuracy. As a result, we generated a network consisting of two layers of nine neurons in each layer. We used the sigmoid activation function. The learning rate was 0.01 (arb. units). In the learning process the classification error was calculated for the training and test set. It served as a criterion to stop the error increase for the test sample. It required approximately 1000–4000 iterations (epochs) for the training set, which took about 15–60 s of calculation time on a PC equipped with a processor like the Intel^®^ Core i3.

To test our feature extraction model in a real-time working HMI we included it in an on-line recognition system controlling a mobile robot. The robot was built from a LEGO NXT Mindstorms^®^ kit. The wireless communication between the robot, the EMG system and the PC running the MyoClass software was implemented through a Bluetooth^®^ interface. The on-line recognition system used the classifier preliminary (off-line) trained for the subject. The software generated 10 Hz commands to the robot corresponding to the recognized gesture.

## 3. Feature Extraction by Spiking Neurons

**Figure 4:**
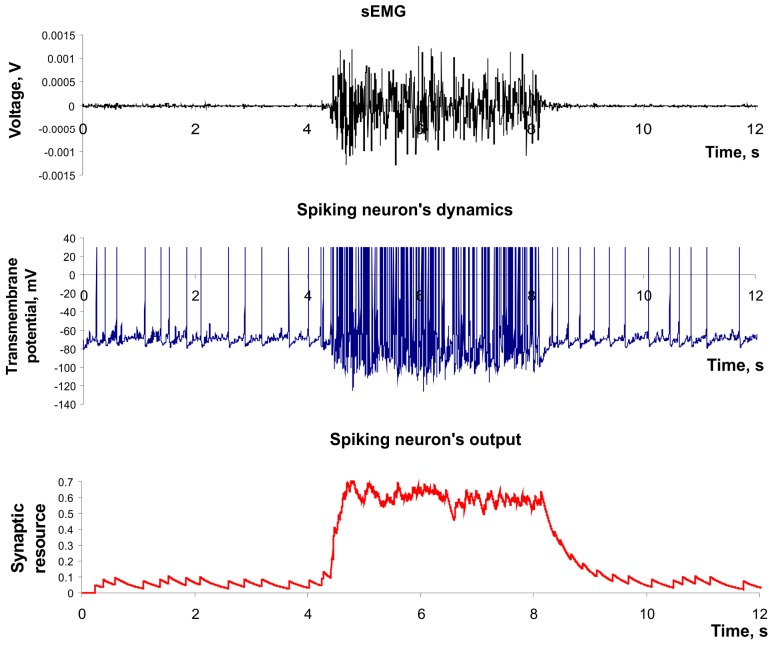
The dynamics of spiking neuron used as a feature extractor. Parameter values: *a* = 0.02*, b =* 0.2*, c =* −65*, d =* 8, *U* = 0.05, *τ_rec_ =* 1 ms, *τ_I_ =* 200 ms, *τ_facil_* = 1 ms, *k* = 2 × 10^6^.

To use the spiking neuron as a features extractor, we assign it as a virtual sensory neuron by applying an external current drive directly to it. For this purpose we introduced the current *I* in Izhekevich’s model (2) as the sum of synaptic current, *I_syn_*, and virtual stimulator current *I_st_*. The synaptic current was calculated as the sum of weighted outputs of the neurons. We set the virtual stimulator current as absolute value of sEMG signal multiplying by a coefficient *k*:

(9) $$I=I_{syn}+I_{st}=\sum_{i}w_{i}g_{i}y_{i}(t)+k\cdot \left|EMG(t)\right|$$

where *w_i_* is a weight of the connection with *i-*th neuron, *g_i_* is the output current scaling coefficient, *y_i_(t)* is the output of the *i*-th neuron, and *EMG(t)* denotes a recorded sEMG signal. We assigned all connected neurons to be inhibitory, therefore *g_i_* was taken negative. We considered neuron output *y(t)* as a continuously changing feature which was monitored at certain discrete time steps defined by the classifier sampling frequency. Figure 4 illustrates the example of the input signal applied to the sensory spiking neuron (e.g., the sEMG signal) and its spiking dynamics in the output (e.g., the feature of sEMG signal). 

Next, we associated each of eight sEMG channels with eight real-time working spiking neurons (2–8). Each 50 ms values of their outputs were projected to the inputs of formal neurons for learning and classification. In simulations, we fixed the following parameters: *a* = 0.02, *b =* 0.2, *c =* –65, *d =* 8, *U* = 0.05, *τ_rec_ =* 1 ms, *τ_I_ =* 200 ms, *τ_facil_* = 1 ms and *k* = 2 × 10^6^ (Figure 4). Primary spiking neurons were not connected with each other. In this model the feature extraction demonstrated a classification accuracy about 1%–3% worse compared with traditional model based on *RMS* (1).

In sEMG we used the array of electrodes located in a ring with a small distance from each other. To improve signal processing efficiency for such closely located electrodes one can realize lateral inhibition in the detection strategy. The lateral inhibition represents a natural way to identify a desired signal and the widely presented in living organisms. In this mechanism neuron excitation (e.g., spikes) via inhibitory inter-neurons suppress the activity of neighboring neurons. In particular, it permits one to increase the contrast and spatial resolution of signal processing in sensory system [15].

**Figure 5:**
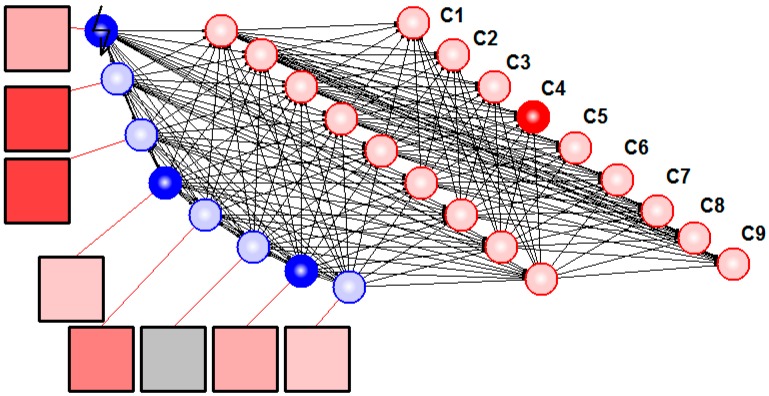
Hybrid neural network recognized sEMG patterns. Squares denote electrodes, blue circles illustrate spiking neurons with synaptic nodes, and red circles are formal neurons. Resulting motion class (No.4 in this case) is determined by the last layer neuron with maximal output (C4).

Furthermore, we applied lateral inhibition in sEMG signal detection. We designed the network so that all sensory spiking neurons had equal inhibitory connections with each other. Figure 5 illustrates resulting hybrid neural network system solving the task of detection and classification of sEMG patterns.

**Figure 6:**
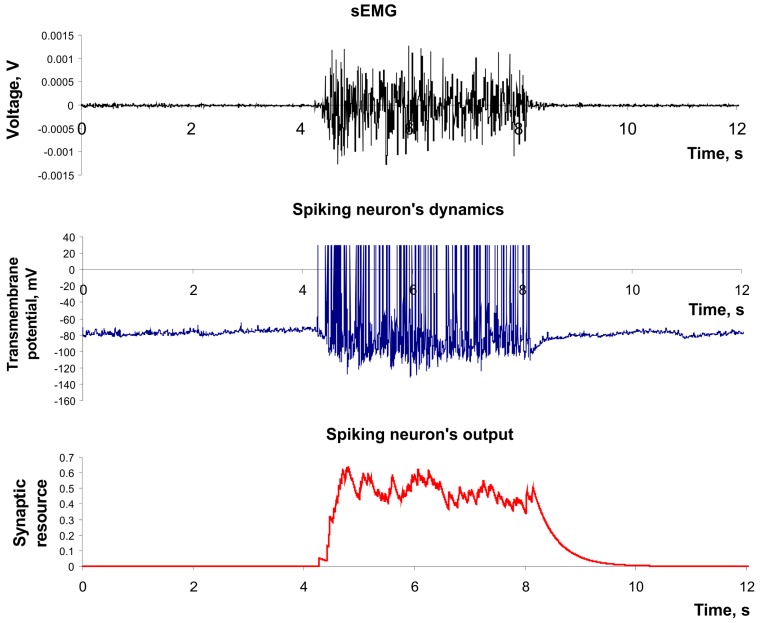
The dynamics of feature extracting neuron included in inhibitory network. Parameter values: *a* = 0.02, *b =* 0.2, *c = –*65, *d =* 8, *U* = 0.05, *τ_rec_ =* 1 ms, *τ_I_ =* 200 ms, *τ_facil_* = 1 ms, *k* = 2 × 10^6^, *w* = 0.5, *g* = 60.

Use of spiking neurons with mutual inhibition as feature extractors permitted us to achieve a classification accuracy comparable with *RMS* (see Table 1 and Table 2). The inhibitory connections had the following parameters: *w* = 0.5, *g* = 60. One can compare the activity of virtual sensory neuron in the network with lateral inhibition (Figure 6) with the activity of equivalent neuron in isolated mode (e.g., in Figure 4). In particular, the background spiking activity (e.g., noise signal) was suppressed in such a network. In addition, the increase of classification accuracy can be explained by suppression of the in-phase activity of different channels because the in-phase component doesn’t carry useful information for the pattern recognition problem.

**Table 1:** The accuracy of classification sEMG-patterns registered by MYO™ Thalmic. Comparison of standard feature of sEMG signal (RMS) spiking neural networks with lateral inhibition (SP_NEURO) used for classification.

Subject	RMS	SP_NEURO	DELTA
Subject 1	91.30%	90.3%	−1.00%
Subject 2	93.60%	95.5%	1.90%
Subject 3	89.40%	90.4%	1.00%
Subject 4	97.85%	98.8%	0.95%
Subject 5	88.40%	91.6%	3.15%
Subject 6	97.25%	95.6%	−1.65%
Subject 7	84.65%	84.7%	0.00%
Subject 8	93.35%	94.5%	1.10%
Subject 9	93.80%	94.1%	0.25%
Subject 10	84.60%	88.0%	3.35%
Mean	91.4%	92.3%	0.9%
Standard deviation	4.7%	4.2%	

**Table 2:** The accuracy of classification sEMG-patterns registered by DELSYS^®^ Trigno™. Notation as in Table 1.

Subject	RMS	SP_NEURO	DELTA
Subject 11	90.5%	89.7%	−0.80%
Subject 12	86.5%	86.9%	0.45%
Subject 13	97.7%	97.0%	−0.70%
Subject 14	93.0%	93.3%	0.30%
Subject 15	85.7%	86.1%	0.40%
Subject 16	92.7%	91.4%	−1.30%
Subject 17	89.0%	90.0%	1.00%
Mean	90.7%	90.6%	−0.1%
Standard deviation	4.2%	3.7%	

Finally, we have illustrated the proposed approach of feature extraction in a simple on-line recognition system. In this example one can navigate a mobile LEGO robot by static hand gestures. Each gesture (except “Rest”) was associated with a command to the robot: “Drive”, “Reverse”, “Forward Right”, “Forward Left”, “Reverse Right”, “Reverse Left”, “Stop”, “Fire” (see Supplementary Materials). The recognition system undergoes a learning procedure for each user. Typically, there were one or two problem gestures recognized uncertainly or mismatched with another gesture. Due to the biological feedback mediated by vision users learned to compensate for such problems apparently by tuning their muscle contraction patterns. If such compensation failed, we excluded these gestures and used a reduced command set to achieve a classifier accuracy of 95%–99%.

## 4. Discussion

We propose a hybrid network model involving bio-mimetic spiking neurons for feature extraction and a formal neural network for pattern classification. Noticeably, the resulting recognition system displayed high accuracy on data recorded from Trigno and MYO systems (see Table 1 and Table 2). Being tested with both systems our model demonstrated equal accuracy despite significant (five-fold) sampling rate differences.

We also illustrated how biologically-based spiking neurons and formal artificial neurons linked in hybrid systems may provide new features to HMI systems enhancing their performance. For example, in our case the activity of virtual neurons in the detector was connected with the generic activity of motor units, and, hence with the activity of motor neurons in the spinal cord. With an increase of the number of registered channels improving the resolution one can expect a better degree of synchronization between individual living neurons and external virtual counterparts. In such an interface the virtual spiking neuron will serve as an artificial expansion of the nervous system that could provide additional functions and degree of flexibility for perspective HMI systems.

## Supplementary Files

Supplementary File 1
